# Chemical Composition of Composite Flour From Okara Powder and Whole Wheat Flour and the Sensory Evaluation of the Biscuits Made From the Composite Flour

**DOI:** 10.1002/fsn3.70450

**Published:** 2025-06-17

**Authors:** Olalekan J. Adebowale, Adeyemi Ayotunde Adeyanju, Tabea Mokhele, Oluwaseun P. Bamidele

**Affiliations:** ^1^ Department of Food Technology The Federal Polytechnic Ilaro Ogun State Nigeria; ^2^ Centre for Innovative Food Research (CIFR) Department of Biotechnology and Food Technology, Faculty of Science University of Johannesburg Johannesburg South Africa; ^3^ Department of Food Science and Microbiology, College of Pure and Applied Sciences Landmark University Omu‐Aran Kwara Nigeria; ^4^ Department of Food Science and Technology University of Venda Thohoyandou South Africa

**Keywords:** amino acids, complexes, functional properties, pasting

## Abstract

The usage of food by‐products and waste has become a significant concern to stakeholders in food processing due to the environmental impact and the waste of resources. The soybean's by‐product is okara, which is rich in protein and other nutrients. The effects of substituting okara powder into whole wheat flour on nutritional qualities, antioxidant activities, and functional and pasting properties of the composite flour were determined using standard procedures. The results showed an increase in the composite flour's nutritional, functional, and antioxidant activities compared to the control (Whole‐wheat flour). The amino acid profile of the composite flour was higher in terms of all essential amino acids than that of the control samples. Okara powder added to whole wheat flour reduced the pasting properties except for the pasting temperature and peak time. The biscuits made with 10%, 20%, and 30% okara powder with whole wheat flour have similar scores to biscuits made with whole wheat flour alone. Compositing okara powder with whole wheat flour may be a raw material for healthy snacks in the confectionery industry.

## Graphical abstract

The study composited whole wheat flour and okara powder, and the addition of okara powder improved the proximate composition of whole wheat flour. The antioxidant activities of the composited flour were higher than the control (whole wheat flour). The sensory panelists scored the biscuit made from composited flour containing 10%, 20% and 30% okara powder, similar to the whole wheat flour.

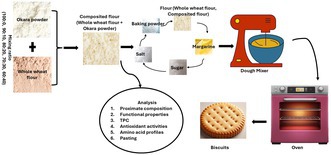

## 1 Introduction

The high cost of wheat importation and shortfalls experienced recently by non‐wheat‐producing countries such as Nigeria and South Africa demand that alternatives be sourced. In Nigeria, imported wheat was priced at an average of $39,464 per ton in 2024, a substantial 5560% rise from 2021 (Alkali 2025). Despite the challenges mentioned, consumers tend to prefer whole wheat food products, possibly because of their unique chemical composition, especially the presence of glutenin and gliadin, which are uncommon proteins in other cereal grains, giving dough elasticity and structure, enabling it to rise and retain its shape (Taylor et al. 2022).

Composite flour technology has been a long‐standing practice that has reduced the burden on non‐wheat‐producing countries' foreign reserves (Adebowale et al. 2009). Developing sustainable solutions for managing food by‐products is one of the main concerns for stakeholders in food processing (Torres‐León et al. 2018; Ibidapo et al. 2020). By‐products like okara contribute to environmental pollution and severe economic losses (Ichikawa et al. 2022). Asian countries like China generated about 2800.00 tons of okara from the soybean curd‐processing sector, and most of it was disposed of in landfills or incinerators (Feng et al. 2021). With the United Nations projected global population increase to 10 billion by 2050, food and nutritional insecurity are imminent, especially in developing countries (Ghosh et al. 2024), and the utilization of okara to make nutritious food may help to combat nutritional insecurity.

Valorised food wastes have the potential to address hunger and nutrient deficiency and promote human health when incorporated into diets (Torres‐León et al. 2018). Okara, a good example of food waste, is the main by‐product of water‐soluble filtrate from soybeans generated after soymilk and/or tofu production (da Silva et al. 2019). Okara is usually disposed of in landfills in Japan and Hong Kong because it is highly perishable (Privatti and Rodrigues 2021). This high level of perishability may have limited its application in food processing (Ichikawa et al. 2022). However, dried okara powder is estimated to have approximately 54% dietary fiber, 33% protein, and 9% fat (all expressed on a dry weight basis) (Mateos‐Aparicio et al. 2010). The efficient usage of okara could lead to economic benefits, a reduction in potential environmental pollution (Wang et al. 2022) and possibly enhanced product (food) sensory quality.

Different studies on the composition of okara powder with other grains have shown that okara powder benefits human health. Adeyanju et al. (2025) studied the effects of adding okara to sorghum flour. They reported an increase in amino acids and other nutritional compositions of the porridge made from the composite flour due to the addition of okara to sorghum flour. Also, AR (2020) substituted wheat flour with okara flour in biscuit production. The author reported that mixed flour biscuits made from okara and wheat flour have higher physicochemical, nutritional, textural, and sensory attributes than biscuits made from wheat flour alone.

As okara is rich in protein, lipids, and dietary fiber, to the best of our knowledge and based on the literature reviewed, there is little to no information on the nutritional, functional, and antioxidant properties of whole wheat flour composite with okara powder. Hence, this study aimed at the chemical composition of composite flour from okara powder and whole wheat flour, and the sensory evaluation of the biscuits made from the composite flour.

## 2 Materials and Methods

### 2.1 Materials and Sample Collection

A single batch of soybeans (20 kg) and the whole wheat grain (10 kg) was purchased at a local market in Thohoyandou, Limpopo Province, South Africa, and stored in a cold room (8°C) in the Pilot Plant in Polypropylene bags before processing. Other biscuit ingredients, including sugar, salt, margarine, milk, baking powder, and vanilla flavor, were purchased at a retail store in Thohoyandou. All chemicals used for the analysis were of analytical grade.

### 2.2 Methods

#### 2.2.1 Processing of Wholewheat Grains and Soybeans Into Flour and Okara Powder

Whole wheat and soybean grains were processed into flour and okara powder. Whole wheat grains were sorted for wholesomeness, washed with potable water, dried in a cabinet dryer at 65°C for 3 h, and milled (three times) in an attrition mill to obtain very fine flour. The method of Adebiyi et al. (2016) was used to produce okara powder from soybean grains. The whole wheat flour and okara powder (Okara) were mixed at 90:10, 80:20, 70:30, and 60:40, while whole wheat flour was the control.

### 2.3 Analyses

#### 2.3.1 Proximate Composition of the Composite Flour

Employing the AOAC method (2005), the proximate composition of the composite flours and the bread was determined (using methods 925, 10, 65.17, 974, 24, and 992.16). The carbohydrate content was calculated by subtracting the percentages of moisture, protein, ash, fat, and fiber from 100%.

#### 2.3.2 Functional Properties of the Composite Flour

The functional properties of the flour samples, encompassing water absorption capacity (WAC), water solubility index, swelling capacity (SC), and bulk density (BD), were determined using a modified method of Adeyanju and Bamidele (2022). The water absorption capacity was determined by introducing 2 g of the sample (W1) into a centrifuge tube and adding 30 mL of water. After vortexing the sample for 10 min, a resting period of another 10 min ensued. Subsequently, the suspension underwent centrifugation for 15 min at a speed of 3000 rpm. After centrifugation, the liquid supernatant was poured off, and the tube containing the residue was weighed (W2). The water absorption capacity was calculated by determining the mL of water absorbed per gram of flour.

$$\mathrm{WAC}=\frac{W2-W1}{\text{Weight of the sample}}\times 100$$

To evaluate swelling capacity and water solubility index, 2 g of flour was measured and placed into a 50 mL centrifuge tube. Subsequently, 30 mL of distilled water was gently mixed with the flour. This mixture was heated in a water bath set at 90°C for 50 min. Throughout the heating process, the slurry was stirred gently to prevent the formation of flour clumps. After 15 min of heating, the centrifuge tube containing the paste underwent centrifugation at a speed of 3000 rpm for 10 min using the Eppendorf centrifuge (5702R Germany). Following centrifugation, the liquid supernatant was immediately decanted. The weight of the sediment was measured and recorded. Subsequently, the moisture content of the sediment gel was determined to establish the dry matter content of the gel.

$$\text{Solubility index}\;\left(\%\right)=\text{weight of}\;\mathrm{dry}\;\text{solid after drying}\times 100$$

$$\text{Swelling power}\;\left(g/g\right)=\frac{\text{weiht of}\;\mathrm{wet}\;\text{mass of sediment}}{\text{weight of}\;\mathrm{dry}\;\text{matter in}\;\mathrm{gel}}$$

An exact 10 g of the flour sample was placed into a 50 mL graduated measuring cylinder to determine the bulk density. The sample was compacted by gently tapping the cylinder on the bench top 10 times from a height of 5 cm until no further volume change occurred. The final volume of the sample was then measured, and the difference between the initial and final volumes was computed and presented as the bulk density in grams per milliliter.

$$\text{Bulk density}\;\left(g/\mathrm{mL}\right)=\frac{\text{Mass of sample}}{\text{volume of sample}}$$

#### 2.3.3 Extraction of Free Phenolic Compounds From the Flour Samples

To extract free phenolic compounds from the flour samples, 1 g was accurately weighed into separate 30 mL beakers, with duplicates for each sample. Subsequently, 10 mL of methanol containing 1% conc. HCl was added to each beaker. The beakers were then covered with foil paper to prevent light exposure and placed on an orbital shaker (Stuart Cole‐Parmer Ltd., India) operating at 200 rpm for 2 h. Following this extraction period, the beakers were removed from the shaker. Next, the samples were centrifuged (LW Scientific, Georgia, United States) at 3500 *g* for 10 min. The supernatant was carefully separated and transferred into a 30 mL centrifuge tube. The residue was subjected to two additional 30‐min extraction cycles to ensure comprehensive extraction. All the supernatants obtained from the different extraction cycles were combined and collected in a 30 mL centrifuge tube. These pooled supernatants were then securely stored at a temperature of −20°C before further analyses. The same extraction procedure was replicated for each flour sample using an alternative solvent, a 70% aqueous acetone solution. The resulting supernatants from these extractions were also stored at −20°C in preparation for subsequent analyses.

#### 2.3.4 Total Phenolic Content

A modification of the method described by Adeyanju and Duodu (2023) was employed to determine the total phenolic content. Separate additions of 0.1 mL of a 1% HCl‐methanol extract of each of the flour samples, blank (extracting solvent), and a phenolic standard (gallic acid; 0.1–0.5 mg/mL) were made to 7 mL of deionized water within individual 15‐mL test tubes in triplicate. Following this, 0.5 mL of Folin–Ciocalteu reagent was introduced to each tube, stirred to ensure proper mixing, and then allowed to incubate at room temperature for a period of 1–8 min. Subsequently, 1.5 mL of a 20% (w/v) aqueous sodium carbonate (Na_2_CO_3_) solution was added to each test tube, and the mixture was subjected to an incubation period lasting 1 h. Thereafter, the absorbance was measured at 765 nm using a UV spectrophotometer (Biochrom Libra PCB 1500, Biochrom Ltd., UK). The results were then expressed as milligrams of gallic acid equivalents (GAE) per gram of the sample.

#### 2.3.5 Antioxidant Assays of the Flour Samples

The ABTS (2,2′‐azino‐bis (3‐ethylbenzothiazoline‐6‐sulfonic acid)) radical scavenging activity of the flour samples was conducted by modifying the procedure outlined by Adeyanju and Duodu (2023). In this process, ABTS (8 mg) was dissolved in 1 mL of deionized water to form solution A. Simultaneously, potassium persulfate (1.32 mg) was dissolved in 1 mL of deionized water to form solution B. By mixing 1 mL of solution A with 1 mL of solution B, the monocation ABTS• + radical was generated. This mixture was then allowed to react in the dark at room temperature for 12–16 h. To prepare a fresh working solution of the ABTS radical cation, 2 mL of the ABTS radical was combined with 58 mL of PBS buffer (pH 7.4). Subsequently, to a 0.2 mL HCl‐methanol extract, 3 mL of the above‐prepared ABTS working solution was added. The resulting mixture was kept in the dark at room temperature for 30 min. Following this incubation, the absorbance was measured at 734 nm using a UV spectrophotometer (Biochrom Ltd. UK). By employing Trolox (water‐soluble vitamin E analogue) as a standard (0, 100, 200, 400, 600, 800 and 1000 μM), the capacity of the sample to scavenge ABTS radicals was assessed and expressed as micromoles of trolox equivalent per gram of the sample (μmol TE/g).

The assessment of DPPH (2,2‐diphenyl‐1‐picrylhydrazyl) radical scavenging activity was carried out using a methodology outlined by Razola‐Díaz et al. (2022), with some modifications. A mother solution was prepared by dissolving 24 mg of DPPH in 100 mL of methanol. Subsequently, a working solution of DPPH was prepared by combining 10 mL of the mother solution with 50 mL of methanol. The absorbance of this working solution was adjusted to around 1.1 at 515 nm using the mother solution. For the assay, 0.2 mL of each 70% aqueous methanol extract was added to 3 mL of the DPPH working solution. This mixture was then placed in the dark at room temperature for 30 min. The absorbance was measured at 515 nm using a UV spectrophotometer (Biochrom Ltd. UK), and the result was quantified as micromole trolox equivalents per gram of the sample (μmol TE/g).

The FRAP (Ferric Reducing Antioxidant Power) assay was conducted according to the protocol outlined by Razola‐Díaz et al. (2022), with slight adjustments. This method operates on the principle of converting an initially colorless ferric‐tripyridyltriazine (Fe^3+^‐TPTZ) complex into a blue ferrous‐colored form facilitated by electron donation in the presence of antioxidants. This procedure combined 100 μL of each extract with 200 μL of deionized water and 2 mL of the FRAP reagent. The mixture was then incubated at 37°C for 30 min, followed by the absorbance measurement at 595 nm. A standard curve of Trolox equivalent (TE) was employed for the assessment, and the result was quantified as micromoles of Trolox equivalents per gram of the sample (μmol TE/g).

### 2.4 Pasting Properties of the Flour Samples

An RVA series 4 (New Port Scientific, Warriewood, NSW, Australia) was used to examine the pasting profile of the composite flour (whole wheat flour plus okara powder). After weighing 3 g of the sample, 25 mL of distilled water were poured into a canister. The canister, which was positioned in the centre of the paddle coupling and subsequently put into the RVA machine, contained a paddle. Pressing the instrument motor tower initiated the measurement cycle using the 12‐min profile. Idle at 50°C for 1 min, heated to 91°C for 3.45 min, and then maintained at 91°C for 2.30 min was the time–temperature regime employed. The sample was cooled to 50°C for 3.45 min, then the temperature was maintained at 50°C for 2 min.

### 2.5 Amino Acid Profiles of the Flour Samples

The amino acid profile of the samples was determined using the methods of Hall and Schönfeldt (2013) and Chavali et al. (2013). The method was reversed‐phase ultra‐pressure liquid chromatography (UPLC).

### 2.6 Biscuit Preparation

The modified method of Momin et al. (2020) was used to prepare the biscuits. The composite flour biscuits were prepared from various combinations of whole wheat flour and okara powder in the ratios of 100:0, 90:10, 80:20, 70:30, and 60:40, respectively. It had ingredients as per 100 g of composite flour, 30 g sugar, 30 g hydrogenated fat (margarine), 2 g of salt, 2 g baking powder, 35 mL of milk, and 0.5 mL vanilla flavor. The margarine and sugar were taken and mixed to a uniform consistency. The flour, required amount of water, baking powder, and sodium bicarbonate were added to the creamed mixture (margarine and sugar) and mixed for 10 min at medium speed in a dough mixer to obtain a homogeneous mixture. The dough was rolled out into a thin sheet of uniform thickness (5 mm) and was cut into 4 cm‐diameter biscuits. The cut pieces were placed over the cleaned tray and transferred into the convective baking oven at 220°C for 10 min till baked. The well‐baked biscuits were removed from the oven, cooled to room temperature, packed in Ziplock bags, and stored at room temperature for sensory analyses.

### 2.7 Sensory Evaluation of the Biscuits

Thirty (30) untrained panel members were used to evaluate the biscuits made from the composite flour (Whole wheat flour + okara powder). Panelists are comprised of students who are regular consumers of biscuits, aged between 20 and 22 years, and they all gave their consent prior to the analysis. The evaluation was conducted in the sensory laboratory with individual assessors in a separate booth. The biscuits were blind‐coded using a 3‐digit code. The attributes, color, texture, aroma, flavor and overall acceptability were evaluated on a 9‐point hedonic scale (1 = dislike extremely to 9 = like extremely).

### 2.8 Statistical Analysis

IBM SPSS statistical software for Windows version 20.0 (IBM, Armonk, NY, USA) was used to analyze the data in triplicate using a one‐way analysis of variance (ANOVA) at a significance threshold of *p* < 0.05. The least significant difference (LSD) was used for post hoc.

## 3 Results and Discussion

### 3.1 Proximate Composition of the Whole Wheat Flour and the Composite Flour

The proximate composition of composite flour from okara and wholewheat flour showed no significant difference (*p* > 0.05) in moisture content with an increase in the addition of okara to wholewheat flour (Table 1). The 100% wholewheat flour has the least moisture content (10.23%), while the moisture contents of the remaining samples ranged between 11.27% and 11.57%. The increase in the moisture content may be attributed to the addition of okara to wholewheat flour. The okara powder may contain higher moisture content, which increases the moisture content of the samples. Adding okara powder to whole wheat flour increases the protein content of all the composite flours. The whole wheat flour (100%) has the least protein content (13.52%), while the protein content of the remaining samples increased with an increase in the percentage of okara powder added. The composite flour (60% wholewheat + 40% okara powder) has the highest protein content (23.54%), followed by the sample that contains 30% okara powder (19.34%).

**TABLE 1:** Proximate composition of composite flour from okara and wholewheat flour (%).

Samples	Moisture	Crude protein	Crude fat	Ash	Crude fiber	Carbohydrate
100%W + 0%OP	10.23^a^ ± 0.6	13.52^a^ ± 0.6	2.61^a^ ± 0.5	0.57^a^ ± 0.1	0.46^a^ ± 0.1	72.61^e^ ± 0.9
90%W + 10%OP	11.27^b^ ± 0.6	15.21^b^ ± 0.5	3.23^b^ ± 0.5	1.12^b^ ± 0.2	2.56^b^ ± 0.2	66.61^d^ ± 0.8
80%W + 20%OP	11.45^b^ ± 0.7	17.56^c^ ± 0.7	5.11^c^ ± 0.6	2.23^c^ ± 0.3	6.77^c^ ± 0.6	56.88^c^ ± 1.1
70%W + 30%OP	11.51^b^ ± 0.7	19.34^d^ ± 0.6	6.86^d^ ± 0.7	2.67^d^ ± 0.4	10.57^d^ ± 0.4	49.05^b^ ± 0.9
60%W + 40%OP	11.57^b^ ± 0.5	23.54^e^ ± 0.7	7.56^e^ ± 0.5	2.88^e^ ± 0.4	15.67^e^ ± 0.7	38.78^a^ ± 0.7

*Note:* Values are *M* ± SD (*n* = 3). The different superscript(s) show significant differences among groups at *p* > 0.05 level by LSD. W is wholewheat flour, and OP is okara flour.

We could say there was a 74.11% increase in protein content between the wholewheat flour and the composite flour that contains 40% okara powder. The increase in the protein content may be attributed to okara powder, which is very rich in protein content (Li et al. 2012). This result is in line with the report of Adeyanju et al. (2025), who reported an increase in the protein content of sorghum enriched with okara powder.

The crude fat content of the samples followed the same pattern as that of protein content. Wholewheat flour has the least crude fat content (2.61%), followed by the sample that contains 10% okara powder (3.23%). The 40% okara powder sample has the highest crude fat (7.56%). There was a 65.48% increase in the crude fat content of whole wheat flour and a sample with 40% okara powder. The increase in the crude fat may be due to adding okara powder to whole wheat flour. Okara powder was obtained from soybean, which is rich in fat. Although okara powder is a by‐product of soybean, it was reported to be rich in crude fat (Mateos‐Aparicio et al. 2010). The ash and the crude fiber contents of the composite flours were higher than that of the whole wheat flour (Table 2). The sample with 40% okara powder has the highest ash (2.88%) and crude fiber (15.67%) contents, while whole wheat flour has the lowest values of ash (0.57%) and crude fiber (0.46%) contents.

**TABLE 2:** Functional properties of composite flour from okara and wholewheat flour.

Samples	WAC (g/g)	SC (%)	BD (g/mL)	WSI (%)
100%W + 0%OP	0.98^a^ ± 0.1	11.23^a^ ± 0.5	0.42^a^ ± 0.1	23.12^e^ ± 0.5
90%W + 10%OP	1.12^b^ ± 0.3	13.46^b^ ± 0.4	0.56^b^ ± 0.2	20.22^d^ ± 0.6
80%W + 20%OP	1.45^c^ ± 0.5	15.67^c^ ± 0.3	0.66^b^ ± 0.1	17.86^c^ ± 0.7
70%W + 30%OP	1.67^c^ ± 0.4	16.11^d^ ± 0.2	1.04^c^ ± 0.1	12.44^b^ ± 0.5
60%W + 40%OP	1.79^c^ ± 0.5	17.86^e^ ± 0.5	1.24^c^ ± 0.2	10.45^a^ ± 0.2

*Note:* Values are *M* ± SD (*n* = 3). The different superscript(s) show significant differences among groups at *p* > 0.05 level by LSD. W is wholewheat flour, and OP is okara flour.

Abbreviations: BD, bulk density; SC, swelling capacity; WAC, water absorption capacity; WSI, water solubility index.

The ash and the crude fiber contents of the composite flour increase with the increasing percentage of added okara powder. As the carbohydrate content of the sample was deduced from the subtraction of other nutritional composition (moisture, protein, crude fat, ash, crude fiber) from 100%, the whole wheat flour has the highest carbohydrate content (72.61%), followed by composite flour that contains 10% okara powder (66.61%). The composite flour with 40% okara powder has the least carbohydrate value (38.78%).

The increase in the carbohydrate value of whole wheat flour may be attributed to the absence of okara powder in the sample. As reported earlier, okara powder is rich in protein and crude fiber. Its addition to whole wheat flour enhanced the nutritional composition of the composite flour, as shown in Table 2. This result is similar to the report of Makinde and Tifu (2021), who reported an increase in the nutritional composition of composite flour from okara and maize flour. It could be said that okara may be a good source of protein and fiber when added to flour at an acceptable percentage, which may need to be determined by the food manufacturer.

### 3.2 The Functional Properties of the Whole Wheat Flour and the Composite Flour

The functional properties of the composite flours (Okara powder and whole wheat flour) are shown in Table 2. There is an increase and significant difference (*p* > 0.05) between the whole wheat and composite flour samples. The whole wheat flour's water absorption capacity (WAC) was the lowest (0.98 g/g), followed by the sample that contained 10% okara powder. The composite flour with 40% okara powder has the highest WAC value (1.79 g/g). Similarly, whole wheat flour's swelling capacity (SC) was the lowest (11.23%), followed by the composite flour with 10% okara powder.

The composite flour with 40% okara powder has the highest swelling capacity (17.86%). The bulk density (BD) followed the same pattern as WAC and SC, with whole wheat flour having the lowest value (0.4 g/mL). The percentage of okara powder to whole wheat flour increases the WAC, SC, and BD. The water solubility index (WSI) was reduced with increased okara powder added to whole wheat flour, with whole wheat flour having the highest value (23.12%), followed by the sample with 10% okara powder (20.22%). The 40% okara powder sample has the lowest WSI value (10.45%). The increase in the functional properties of the composite flour may be attributed to the addition of okara powder, which is rich in crude fiber (Li et al. 2012). The WAC is the amount of water that binds with flour to get the desired consistency for product preparation (Awuchi et al. 2019). The protein and crude fiber contents have positive and negative effects on the WAC of composite flour (Godswill 2019). The increase in WAC and SC of the composite flour samples may be attributed to increased crude fiber due to the addition of okara powder. Crude fibers are known to have high WAC because of their cellulose content and hollow cavities, which allow high water retention during processing (Godswill 2019). It is known that bulk density (BD) helps measure how heavy a food sample is and determine the type of packaging material such a sample requires (Eisenbies et al. 2019). The increase in the BD of the composite flour may be due to the addition of okara powder to whole wheat flour.

The presence of high crude fiber in okara powder may be responsible for the high BD value of the samples. The decrease in the WSI may also be attributed to high crude fiber, which may reduce the solubility of the sample in water. These results align with the report of Uzo‐Peters and Ola (2020), who reported an increase in WAC, SC and BD of composite flour between sorghum and okara powder and a decrease in WSI of the composite flour due to crude fiber and insoluble protein in okara powder.

### 3.3 Total Phenolic Content and Antioxidant Activities of Whole Wheat Flour and the Composite Flour

Table 3 shows the total phenolic content (TPC) and antioxidant activity (ABTS, DPPH, and FRAP) of the whole wheat flour and the composite flours (Whole wheat flour + okara powder). The TPC of the whole wheat flour was the lowest (19.23 mg GAE/g) among the whole samples. The addition of okara powder to whole wheat flour increases the TPC of the composite flour. The increase in TPC increased with the percentage of okara powder added to whole wheat flour. Whole wheat flour with 10% okara powder has the lowest value (24.56 mg GAE/g) among the composite flours, followed by whole wheat flour with 20% okara powder (45.23 mg GAE/g). Whole wheat flour with 40% okara powder has the highest TPC (62.45 mg GAE/g). The increase in the TPC of the composite samples may be attributed to adding okara powder to whole wheat flour. Okara powder has been reported to have high phenolic compounds (Spréa et al. 2024). Spréa et al. (2024) reported that okara powder is rich in phenolic compounds, mainly isoflavones such as catechol group, genistin, genistein, and kaempferol. An increase in TPC may lead to increased antioxidant activities in food samples.

**TABLE 3:** Total phenolic content and antioxidant activities of composite flour from okara and whole wheat flour.

Samples	TPC (mg GAE/g)	ABTS (μmol TE/g)	DPPH (μmol TE/g)	FRAP (μmol TE/g)
100%W + 0% OP	19.23^a^ ± 0.3	7.34^a^ ± 0.1	8.56^a^ ± 0.5	3.23^a^ ± 0.5
90%W + 10% OP	24.56^b^ ± 0.5	8.87^b^ ± 0.5	9.98^b^ ± 0.5	4.34^b^ ± 0.5
80%W + 20% OP	45.23^c^ ± 0.6	9.92^c^ ± 0.5	10.21^c^ ± 0.5	5.57^c^ ± 0.5
70%W + 30% OP	56.17^d^ ± 0.5	11.12^d^ ± 0.5	12.56^d^ ± 0.5	6.78^d^ ± 0.5
60%W + 40% OP	62.45^e^ ± 0.7	15.45^d^ ± 0.5	16.89^d^ ± 0.5	8.89^e^ ± 0.5

*Note:* Values are *M* ± SD (*n* = 3). The different superscript(s) show significant differences among groups at *p* > 0.05 level by LSD. W is wholewheat flour, and OP is okara powder.

**TABLE 4:** Amino acid profile of whole wheat flour and composite flour from whole wheat and okara powder (mg of amino acid per gram of protein).

Amino acid	100% W + 0%OP	90% W + 10%OP	80% W + 20%OP	70% W + 30%OP	60% W + 40%OP
Essential amino acids					
Histidine	15.1 ± 0.9a	15.3 ± 1.2b	15.6 ± 1.4c	15.9 ± 2.1d	16.2 ± 2.5e
Isoleucine	23.5 ± 0.7a	24.1 ± 1.6b	24.5 ± 1.7c	25.0 ± 2.5d	25.5 ± 3.1e
Leucine	53.0 ± 0.9a	54.1 ± 2.5b	55.3 ± 2.4c	56.0 ± 3.2d	57.0 ± 5.3e
Lysine	18.5 ± 0.8a	20.2 ± 2.9b	21.9 ± 2.3c	23.6 ± 2.3d	25.3 ± 3.5e
Methionine	10.5 ± 0.8a	10.8 ± 1.6a	11.1 ± 1.5b	11.4 ± 2.1b	11.7 ± 1.5c
Phenylalanine	38.1 ± 1.2a	38.5 ± 2.5b	39.0 ± 2.5c	39.5 ± 3.1d	40.0 ± 1.7e
Threonine	20.2 ± 0.9a	20.5 ± 3.4a	21.0 ± 1.8b	21.5 ± 2.3c	22.0 ± 1.6d
Tryptophan	6.1 ± 0.5a	6.3 ± 0.9b	6.6 ± 0.5c	6.9 ± 0.7d	7.2 ± 2.2e
Valine	28.1 ± 1.3a	28.7 ± 2.6a	29.4 ± 2.7b	30.1 ± 2.5c	30.8 ± 5.2d
Total essential amino acids	213.1 ± 5.1a	218.5 ± 7.3b	224.4 ± 4.2c	229.9 ± 5.6d	235.7 ± 5.6e
Non‐essential amino acids					
Alanine	35.1 ± 3.1a	36.2 ± 3.2b	37.1 ± 4.3c	38.2 ± 3.5d	39.1 ± 4.2e
Arginine	42.1 ± 3.5a	44.5 ± 5.2b	47.2 ± 5.6c	49.5 ± 4.1d	52.2 ± 5.2e
Aspartic acid	60.1 ± 4.3a	63.2 ± 5.6b	66.1 ± 2.1c	69.1 ± 3.3d	72.3 ± 4.2e
Cysteine	8.5 ± 0.7a	8.8 ± 0.6b	9.1 ± 0.9c	9.4 ± 0.8c	9.7 ± 0.9d
Glutamic acid	120.0 ± 12.2e	118.5 ± 11.3d	117.0 ± 9.7c	115.5 ± 10.6b	114.013.5a
Glycine	22.3 ± 2.5a	23.5 ± 2.7b	25.0 ± 2.1c	26.5 ± 2.4d	28.0 ± 0.2e
Proline	30.2 ± 3.4d	29.5 ± 3.2c	29.0 ± 2.5b	28.5 ± 3.1b	28.1 ± 0.5a
Serine	25.1 ± 1.4a	25.5 ± 2.1b	26.1 ± 1.9c	26.5 ± 3.2d	27.1 ± 0.8e
Tyrosine	16.1 ± 0.9a	16.5 ± 0.9b	17.2 ± 2.1c	17.5 ± 2.3d	18.2 ± 0.6e
TNEAA	359.5 ± 6.1a	366.2 ± 7.2b	373.8 ± 9.2c	380.7 ± 8.6d	388.7 ± 9.4e

*Note:* Values are *M* ± SD (*n* = 3). The different superscript(s) show significant differences among groups at *p* > 0.05 level by LSD. WWF is wholewheat flour, and OP is okara powder.

The composite samples' antioxidant activities (ABTS, DPPH, and FRAP) increased with the percentage of okara powder added to whole wheat flour. The ABTS value of the whole wheat flour was the lowest (7.34 μmol TE/g), and whole wheat flour with 40% okara powder had the highest ABTS value (15.45 μmol TE/g). There is about a 110% increase in the scavenging power of whole wheat flour and composite flour (60% whole wheat flour +40% okara powder). The DPPH value of the samples followed the same pattern as the ABTS value. The DPPH value of the whole wheat flour was the lowest (8.56 μmol TE/g), followed by whole wheat flour with 10% okara powder (9.98 μmol TE/g). Whole wheat flour with 40% okara powder has the highest DPPH value (16.89 μmol TE/g). There is an increase of 97.31% in the scavenging power of whole wheat flour and composite flour (60% Whole wheat +40% okara powder). FRAP (Ferric Reducing Antioxidant Power) values increase with the percentage of okara powder added to whole wheat flour. The highest FRAP value was obtained from composite flour containing 40% okara powder (8.89 μmol TE/g), while the lowest was from whole wheat flour (3,23 μmol TE/g). The increased scavenging power of all the composite flour may be attributed to adding okara powder to whole wheat flour. As reported earlier, okara powder is rich in phenolic compounds, and an increase in phenolic compounds has been attributed to the increased scavenging power of the samples. Although isoflavones comprise the majority of okara phytochemicals, phenolic compounds or other flavonoids are primarily responsible for okara's antioxidant potential (Lee et al. 2020). This is because, in contrast to the hydroxyl or keto groups of isoflavones, these compounds (Phenolic compounds) include catechol or pyrogallol groups, which have more reducing power and radical scavenging action.

### 3.4 Amino Acids Profile of the Samples

Table 2 shows the amino acid profiles of okara powder and whole wheat flour. The findings show that okara powder and whole wheat flour differ significantly in their amino acid composition. Essential amino acids (EAA) are less abundant in whole wheat flour (WWF) than in composite flour. The higher amount of essential amino acids in the composite flour may be attributed to the addition of okara powder, which was reported to contain more essential amino acids (Adeyanju et al. 2025). The increase in the percentage of okara powder added to whole wheat flour increases the EAA. Among the EAA, Leucine was the highest, with 53.0 mg/g protein in whole wheat flour and 57.0 mg/g protein in composite flour (60% WWF + 40% Okara Powder) (Table 4).

Tryptophan has the lowest value among the EAA, with 6.1 mg/g protein in WWF and 7.2 mg/g protein in composite flour whole (60% WWF + 40% Okara Powder). There is a significant difference (*p* < 0.05) in the total essential amino acids (TEAA) of WWF and composite amino acids. The TEAA of the WWF was the lowest (213.1 mg/g protein), followed by the composite flour with 10% okara powder (218.5 mg/g protein). The highest TEAA was obtained in composite flour that contained 40% Okara powder (235.7 mg/g protein). Cereals are believed to be deficient or low in some essential amino acids (Threonine, Leucine and Histidine) (Tomičić et al. 2022). These essential amino acids were improved in the composite flour due to the addition of okara powder to whole wheat flour.

Regarding total non‐essential amino acids (TNEAA), composite flours are significantly (*p* < 0.05) higher in total non‐essential amino acids compared to whole wheat flour. The WWF have the lowest TNEAA (359.5 mg/g protein), and composite flour with 40% okara powder has the highest value (388.7 mg/g protein). The glutamic acid in WWF was higher (120.0 mg/g protein) than in the composite flours, with the composite flour containing 40% Okara powder having the lowest glutamic acid value (114.0 mg/g protein). The decrease in the glutamic acid values of the composite flour may be attributed to adding Okara powder to the WWF. Whole wheat flour has been reported to have a higher amount of glutamic acid when compared to refined wheat flour (Gómez et al. 2020). Okara is a by‐product of soybeans that may be responsible for the lower amount of glutamic acid. It could be said that adding okara powder to WWF increases the total non‐essential amino acids of the composite flour, and an increase in the percentage of okara powder added to WWF increases the TNEAA of the composite flour.

Compositing wheat flour with okara is not necessarily new, but compositing whole wheat flour with okara flour is not shared. Okara powder helps improve the amino acids (both essential and non‐essential) of the composite flour, which means eating products such as bread, biscuits, and other confectioneries made from the composite flour will improve the consumer's health. Also, for protein‐energy malnutrition, the composite flour may help to address this challenge in most parts of the world. These results align with the findings of Adeyanju et al. (2025), who reported an increase in the amino acid content of composite sorghum and okara powder and the fermented product.

### 3.5 Pasting Properties

Table 5 displays the pasting characteristics of whole wheat flour (WWF) and composite flour (WWF + Okara powder). The pasting qualities of WWF and whole wheat flour–okara composite flour differ significantly (*p* < 0.05), according to the pasting viscosity (PV) data. The highest PV (3450.12 cP) was shown by 100% whole wheat flour, indicating its high starch content and notable swelling potential. Adding okara to whole wheat resulted in a considerable decrease in viscosity. The corresponding cP values for composite flour with 10%, 20%, 30%, and 40% okara powder are 3300.2, 3150, 3000, and 2850.6. The addition of okara powder to WWF may be the cause of the PV decline.

**TABLE 5:** Pasting properties of whole wheat flour and composite flour from whole wheat and okara powder.

Samples	Peak viscosity (cP)	Trough viscosity (cP)	Breakdown viscosity (cP)	Final viscosity (cP)	Setback viscosity (cP)	Pasting temperature (°C)	Peak time (mins)
100% W + 0% OP	3450.6 ± 12.0e	2100.3 ± 12.2e	1350.3 ± 9.2e	3100.6 ± 8.2e	1000.3 ± 10.2e	85.5 ± 1.1a	24.5 ± 0.3a
90% W + 10% OP	3300.4 ± 12.2d	2000.2 ± 11.3d	1300.2 ± 9.7d	2950.6 ± 7.8d	950.4 ± 8.3d	86.0 ± 1.6b	29.2 ± 0.5b
80% W + 20% OP	3150.6 ± 12.3c	1950.1 ± 9.8c	1200.5 ± 11.3c	2800.8 ± 9.7c	850.4 ± 6.8c	86.5 ± 1.5c	29.6 ± 0.8b
70% W + 30% OP	3000.6 ± 10.3b	1850.2 ± 9.4b	1150.4 ± 8.7b	2650.7 ± 9.2b	800.5 ± 7.4b	87.0 ± 1.8d	29.9 ± 0.9b
60% W + 40% OP	2850.6 ± 12.2a	1750.1 ± 10.1a	1100.5 ± 9.1a	2500.5 ± 10.2a	750.4 ± 5.6a	87.5 ± 1.8e	29.9 ± 0.8b

*Note:* Values are *M* ± SD (*n* = 3). The different superscript(s) show significant differences among groups at *p* > 0.05 level by LSD. W is whole wheat flour, OP is Okara Powder.

During pasting, leached‐out amylose may form complexes with lipids (Huang et al. 2020) or other okara components, such as dietary fiber, reducing PV. Although the proteins and lipids in okara can obstruct the gelatinization process, the high fiber content may also compete for water, decreasing the amount of water available for starch granule swelling.

The observed holding strength (Trough) values reveal significant variations across the samples (WWF and the composite flour). Whole wheat flour (100%) exhibited the highest holding strength at 2100.2 cP, indicating a strong viscosity and gel stability under prolonged heating and mechanical stress.

This value was reduced by including okara, as seen in WWF: Okara powder 90:10, 80:20, 70:30, and 60:40, with strengths of 2000.2, 1950.6, 1850.4, and 1750.6 cP, respectively. This reduction suggests that okara powder decreased the viscosity of the composite flour.

WWF's breakdown viscosity (BV) was higher than the composite samples. Breakdown viscosity refers to how much a starch paste disintegrates or loses its viscosity when subjected to heat and shear stress (Pacheco et al. 2024). WWF has a higher breakdown viscosity, signifying more stability against disruption of the starch during pasting. Composite flour has a lower BV than WWF, and the decrease in BV of the composite flour may be attributed to the okara powder added to WWF, which disrupts the stability of starch during pasting. As okara powder is rich in crude fat, dietary fiber, and protein (Li et al. 2012), all these components tend to disrupt the stability of the composite flour, leading to lower BV.

All the samples' final viscosity (FV) followed the same pattern as PV. Final viscosity is the viscosity of starch paste at the end of a cooling process after heating, indicating its ability to form a gel and its stability once cooled down (Li et al. 2019). The WWF has a higher FV (3100.6 cP), indicating strong gel formation, which is a desirable trait for products requiring thickness and stability. The composite flour showed a lower FV, and the FV decreased with the percentage of okara powder added to WWF. The decrease in the FV of the composite flour may be due to the addition of okara powder to WWF, leading to less thickening and possibly a waterier gel consistency. The lower FV of the composite flour may be attributed to the action of high crude fiber and crude fats in okara powder that may prevent the gel stability after cooling (Adeyanju et al. 2025).

One important indicator of the retrogradation tendency of starches that affects the final product's texture and stability is the setback viscosity (SV). When okara powder is added to whole wheat flour, the setback results show a substantial decrease. The WWF has the highest SV (1000.3 cP), followed by composite flour with 10% okara powder (950.4 cP). The lowest SV (750.4 cP) was recorded for composite samples with 40% okara powder, which showed that the higher the percentage of okara added to WWF, the lower the SV. SV helps measure the retrogradation tendency of a paste after cooling. The lower SV of the composite samples means they have a lower tendency to retrograde after cooling. It could be said that okara powder lowers the composite samples' retrogradation by preventing the starch granules' re‐association to form a junction zone.

The pasting temperature and peak time showed the effects of okara powder on whole wheat flour. The WWF has a lower pasting temperature (85.5°C) and peak time (24.5 min). Adding okara powder to WWF slightly increases the pasting temperature from 85.5°C to 86.5°C and 87.5°C, respectively. The slight increase in the pasting temperature may be attributed to the coating of the starch granules by lipids in okara powder during pasting, which prevents water absorption by the starch granules. The peak time increased from 24.5 min to 29.2, 29.6, 29.9, and 29.9 min, respectively. The increase in the peak time may be attributed to the action of okara powder components, preventing the rapid absorption of water by the starch granules. Most of the findings of this study were in line with the report of Adeyanju et al. (2025), except for the peak time, which they reported a decrease with an increase in okara added to sorghum flour.

### 3.6 Sensory Evaluation Report of the Biscuit

**FIGURE 1:**
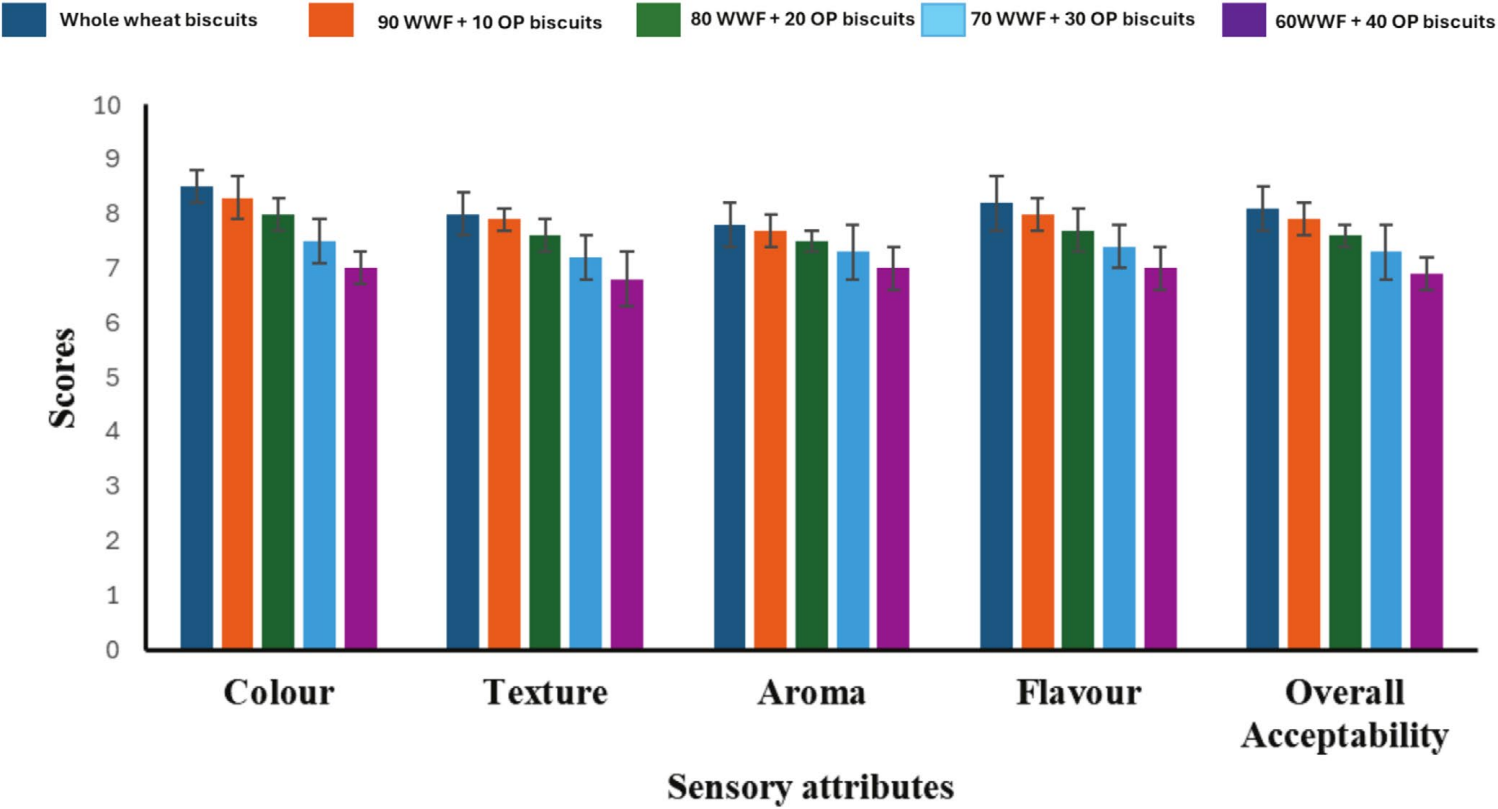
Sensory evaluation of biscuit samples made from whole wheat flour and the composite flour (whole wheat flour + okara powder).

The sensory evaluation report of the biscuit made from the composite flour (whole wheat flour + okara powder) is shown in Figure 1. The color of the biscuit made from whole wheat flour was rated higher at 8.50, followed by biscuits made from composite flour containing 10% okara powder (8.30). The colors of other biscuits made from composite flour containing 20%, 30%, and 40% okara flour were 8.00, 7.50, and 7.00, respectively. The scoring of the biscuits decreases with an increase in the percentage of okara powder added to whole wheat flour. The texture, aroma, flavor, and overall acceptability follow the same pattern as the color. The whole wheat biscuit has the highest values for texture (8.00), aroma (7.80), flavor (8.20) and overall acceptability (8.10). The biscuit from composite flour with 40% okara powder has the lowest score for texture (6.80), aroma (7.00), flavor (7.00) and overall acceptability (6.90).

The decrease in the score of the biscuit made from composite flour may be due to the addition of okara powder to the whole wheat flour. As okara powder is rich in fiber, a high amount of fiber in the biscuit may be responsible for the lower score recorded for texture. The aroma scores for the biscuits were not bad because all the panelists gave similar scores with no significant (*p* > 0.05) differences among the first three biscuit samples and the last two biscuit samples.

The flavor score of the biscuit showed no significant difference (*p* > 0.05) among the biscuit samples made from composite flour containing 10% and 20% okara flour compared to biscuit samples made from WWF. Also, the biscuit samples from composite flour containing 30% and 40% okara powder have no significant difference (*p* > 0.05). The overall acceptability of all the biscuits showed that panelists preferred biscuits made from whole‐wheat flour. Among the biscuits made from composite flour, biscuits containing 10% okara powder were preferred, while biscuits from 40% okara composite flour were less preferred. The results of this study were like the results of Momin et al. (2020), who reported that the sensory panelists preferred biscuits made from wheat flour compared to biscuits made from composite wheat flour and okara flour. It could be said that adding up to 30% okara powder to whole wheat flour may have little or no difference in the sensory quality of products.

## 4 Conclusion

This study evaluates the effects of adding okara powder to whole wheat flour at different percentages. From the results, it could be said that an increase in the percentage of okara powder to wheat flour increases the nutritional value, amino acid profile and the functional properties of the composite flour. Also, the total phenolic content and the antioxidant activities of the composite flour are higher than those of whole wheat flour, which indicates that the composite flour has higher health benefits when processed into food. The sensory evaluation of the composite biscuits shows an acceptable level of 30% of okara powder to whole wheat flour.

## Author Contributions

**Olalekan J. Adebowale:** methodology (equal), validation (equal), writing – original draft (equal). **Adeyemi Ayotunde Adeyanju:** data curation (equal), methodology (equal), writing – original draft (equal). **Tabea Mokhele:** data curation (equal), formal analysis (equal), writing – original draft (equal). **Oluwaseun P. Bamidele:** conceptualization (equal), data curation (equal), methodology (equal), writing – review and editing (equal).

## Conflicts of Interest

The authors declare no conflicts of interest.

## Data Availability

Data will be made available at a reasonable request.
